# FOXM1b, which is present at elevated levels in cancer cells, has a greater transforming potential than FOXM1c

**DOI:** 10.3389/fonc.2013.00011

**Published:** 2013-01-31

**Authors:** Andy K. Y. Lam, Adaline W. L. Ngan, Man-Hong Leung, Davis C. T. Kwok, Vincent W. S. Liu, David W. Chan, Wai Y. Leung, Kwok-Ming Yao

**Affiliations:** ^1^Department of Biochemistry, The Li Ka Shing Faculty of Medicine, The University of Hong KongHong Kong SAR, China; ^2^Department of Obstetrics and Gynaecology, The Li Ka Shing Faculty of Medicine, The University of Hong KongHong Kong SAR, China

**Keywords:** FOXM1b, FOXM1c, transforming potential, MEK1, proteolytic processing

## Abstract

The forkhead box (FOX) M1 transcription factor is required to maintain the proliferation of cancer cells. Two transcriptionally active isoforms of FOXM1, FOXM1b and FOXM1c, have been identified, but their functional differences remain unclear. FOXM1c is distinguished from FOXM1b by an extra exon (exon Va) that contains an ERK1/2 target sequence. Based on a literature search and quantitative PCR analysis, we concluded that FOXM1b is the predominant isoform that is overexpressed in cancers. The further characterization of FOXM1b and FOXM1c revealed two interesting differences. First, FOXM1b exhibited a higher transforming ability than FOXM1c in a soft agar assay. Second, the transactivating activity of FOXM1c, but not that of FOXM1b, was sensitive to activation by RAF/MEK/MAPK signaling. Importantly, the MEK1 activation of FOXM1c was associated with proteolytic processing to generate short forms that might represent constitutively active forms missing the N-terminal inhibitory domain; in contrast, the proteolytic processing of FOXM1b did not require MEK1 activation. Our findings suggest that FOXM1b is functionally more active. These results provide novel insights into the regulation of FOXM1 activity and its role in tumorigenesis.

## INTRODUCTION

Forkhead box (Fox) M1 is a proliferation-specific transcription factor that is ubiquitously expressed in embryonic tissues and cultured cells (Wierstra and Alves, 2007;Kalin et al., 2011). FoxM1 knockout mice died *in utero* between E13.5 and E16.5, and the developing liver and heart exhibit structural abnormalities (Korver et al., 1998;Krupczak-Hollis et al., 2004). Cells with enlarged polyploid nuclei were found in the abnormal tissues, providing the first indication that FoxM1 function may be required for the proper coupling of the S and M phases of the cell cycle. Importantly, FoxM1^-^^/^^-^ MEFs and FOXM1-depleted cancer cells have difficulty undergoing mitosis and exhibit chromosomal instability and polyploidy (Laoukili et al., 2005;Wang et al., 2005;Wonsey and Follettie, 2005). Microarray, chromatin immunoprecipitation (ChIP), and more recently, ChIP-seq analyses have identified important G2/M-specific genes, such as cyclin B1, Cdc25B, CenpA, and Aurora B, as direct targets of FoxM1 (Laoukili et al., 2005;Wang et al., 2005;Wonsey and Follettie, 2005;Chen et al., 2012). These findings support the hypothesis that FoxM1 plays a critical role in mitosis.

Considering the critical role of FOXM1 in promoting mitosis, it is not surprising that the level of this protein is upregulated in various human cancers. Indeed, the depletion of FoxM1 in various mouse models suppresses cell division and thus tumor formation (Wierstra and Alves, 2007;Kalin et al., 2011). Although FoxM1 expression is initiated before S phase entry, its transcriptional activity is suppressed until the G2/M phase when the FoxM1 protein becomes highly phosphorylated (Korver et al., 1997;Leung et al., 2001;Laoukili et al., 2007). FoxM1 phosphorylation is promoted by mitogenic signals. In cancer cells, which are usually subjected to enhanced mitogenic signals, elevated FOXM1 levels were found to promote the G1/S transition and to suppress senescence (Wang et al., 2008;Anders et al., 2011). FOXM1 upregulation was also shown to promote cancer initiation and maintenance by inducing genomic instability (Gemenetzidis et al., 2009,^2010^;Teh, 2012). In gliomas, FOXM1 was recently shown to interact with β-catenin to promote the nuclear translocation of β-catenin to activate the expression of WNT target genes in glioma stem cells, leading to their self-renewal and tumorigenesis (Zhang et al., 2011).

The various effects of FOXM1 are thought to be mediated by two transcriptionally active isoforms, FOXM1b and FOXM1c (Wierstra and Alves, 2007). The only difference between FOXM1c and FOXM1b is the presence of exon Va, which contains an ERK1/2 target sequence, in the c isoform (Ma et al., 2005). In addition to exon Va, the transcriptionally inactive isoform FOXM1a contains exon VIIa, which confers an inhibitory effect on transcription. RT-PCR and RNase protection analyses indicate that FOXM1c is ubiquitously expressed in various primary and secondary cell lines and in neonatal tissues rich in mitotically active cells, whereas FOXM1b is the major isoform expressed in the skin and testes (Yao et al., 1997;Teh et al., 2002;Ma et al., 2005). The analysis of the more ubiquitous c isoform in cultured cells byWierstra and Alves (2006a,c) demonstrated that FOXM1c is kept inactive by internal inhibitory domains and by RB binding. However, most overexpression analyses in animal models have been performed using FOXM1b (Kalin et al., 2011). Hitherto, the parallel comparison of FOXM1c and FOXM1b function in either cell or animal models has not been performed, apart from the early studies showing that both isoforms are transcriptionally active in transient reporter assays (Ye et al., 1997;Leung et al., 2001). The a isoform is not conserved in mouse and its physiological significance remains unclear (Ma et al., 2005).

## FOXM1b, THE ISOFORM UPREGULATED IN CANCER CELLS, HAS A HIGHER TRANSFORMING POTENTIAL

**FIGURE 1:**
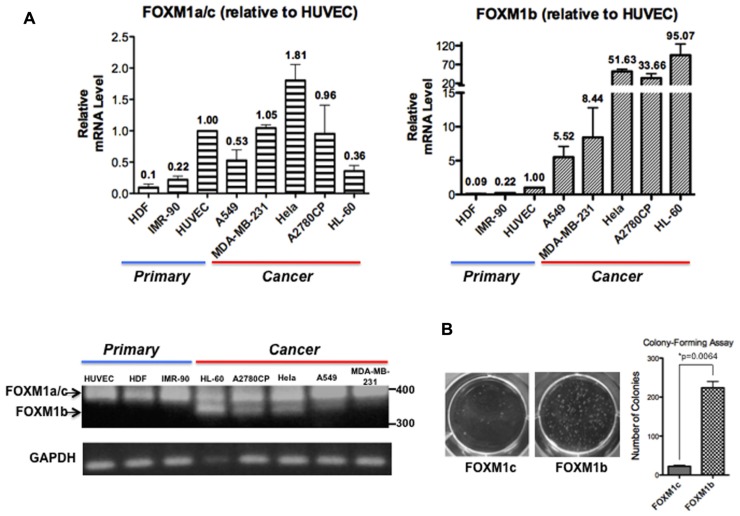
**FOXM1b has higher expression levels and transforming potential in cancer cells**. **(A)** FOXM1 mRNA levels of three primary and five cancer cell lines were determined by RT-PCR and real-time qPCR analyses. Using primers flanking exon Va, RT-PCR analysis led to the amplification of 323 and 368-bp products for FOXM1b and non-FOXM1b (i.e., FOXM1a and FOXM1c) transcripts, respectively. For real-time qPCR analysis, predesigned ABI TaqMan probes for FOXM1b (assay ID: Hs01080645_m1) and FOXM1a/c (assay ID: Hs01073587_m1) transcripts were used to determine their levels against glyceraldehyde 3-phosphate dehydrogenase (GAPDH) transcripts. Relative FOXM1 expression levels are shown as mean ± SE, with the HUVEC level set as 1.00. **(B)** Soft agar colony-forming assay performed using A2780CP cells revealed that the transforming potential of FOXM1b is 10-fold greater than that of FOXM1c. *P = 0.0064.

Analyses of FOXM1 expression in tumor samples of diverse origins have consistently demonstrated a tight correlation between FOXM1 upregulation and enhanced tumorigenic potential but most studies have not addressed whether FOXM1b and FOXM1c are differentially regulated. After reviewing PubMed articles identified using FOXM1 as a keyword, we identified eight studies in which the FOXM1b and FOXM1c transcript levels were distinguished by RT-PCR or qPCR analyses (Teh et al., 2002,^2012^;Liu et al., 2006;Gemenetzidis et al., 2009;Nakamura et al., 2010;Zhang et al., 2011;Dibb et al., 2012;Newick et al., 2012). In these studies, FOXM1b was reported to be the predominant isoform overexpressed in cancer cells, whereas FOXM1c is ubiquitously expressed in both normal and cancer cells. To validate this result, we subjected three human primary cell lines [HDFs (skin), HUVECs (blood vessel), and IMR-90 cells (lung)] and five cancer cell lines [A549 (lung), MDA-MB-231 (breast), HeLa (cervix), A2780CP (ovary), and HL-60 (blood)] to RT-PCR and real-time qPCR analyses. As shown in **Figure 1A**, using primers flanking exon Va, the b-specific product of 323 bp was detectable in the cancer cell lines but not in the primary cell lines, whereas all samples showed the 368 bp c- or a-specific fragment. With more sensitive qPCR assays, two TaqMan probes (from ABI) that detect the b-specific and non-b (i.e., FOXM1a and FOXM1c) transcripts, respectively, were employed. When compared with the levels in HUVECs, the levels of FOXM1b mRNA remained low in the primary cell lines but increased by 5.5-fold (e.g., A549) to 95-fold (HL-60) in cancer cells (**Figure 1A**). In contrast, the levels of the FOXM1a and FOXM1c transcripts did not increase much in the cancer cell lines. If the increased levels of the b isoform in cancer cells were solely due to changes in proliferative potential and conditions of culturing, we would expect corresponding changes of all isoforms. However, the varying extent of increases in FOXM1c levels in the different cancer cell lines may partially reflect a cell/tissue type effect and extensive analysis of more cell lines of different tissues would be required to distinguish cell or tissue type specific effect. As a first attempt to determine whether the b isoform, the level of which is elevated in cancer cells, has a transforming potential similar to that of the c isoform, we ectopically expressed FOXM1b or FOXM1c in A2780CP cells and performed a soft agar colony-forming assay. FOXM1b exhibited a transforming potential 10-fold greater than that of FOXM1c (**Figure 1B**).

## FOXM1c, BUT NOT FOXM1b, IS ACTIVATED BY RAF/MEK/MAPK SIGNALING

**FIGURE 2:**
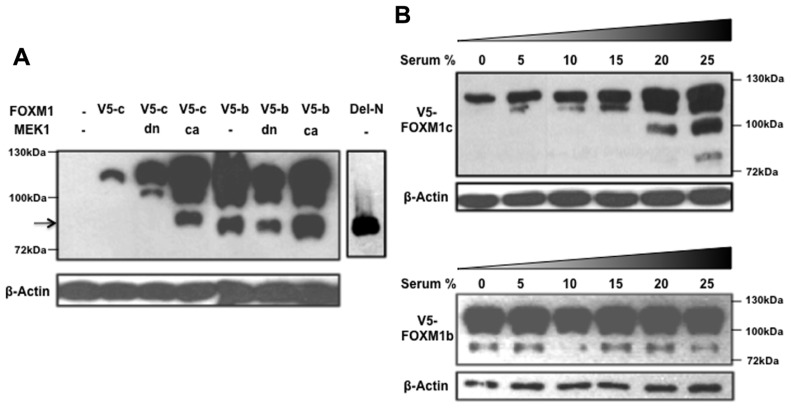
**RAF/MEK/MAPK signaling stimulates the proteolytic processing of FOXM1c**. **(A)** HEK293 cells were cotransfected with V5-tagged FOXM1c (V5-c) or FOXM1b (V5-b) cDNA in the presence or absence of dominant negative (dn) or constitutively active (ca) MEK1 cDNA. Immunoblot analysis using an anti-V5 antibody revealed that proteolytic processing of FOXM1c is enhanced with activated RAF/MEK/MAPK signaling. The arrow indicates a truncated form of V5-tagged FOXM1 that migrated with similar mobility as the Del-N form of FOXM1c. The proteolytic processing of FOXM1b appears constitutive and is unaffected by RAF/MEK/MAPK signaling. **(B)** V5-tagged FOXM1c- or FOXM1b-transfected HEK293 cells were subjected to treatment with medium containing different amounts of serum for 48 h before their harvest for immunoblot analysis. The proteolytic processing of FOXM1c is enhanced with increases in the serum concentration whereas FOXM1b processing is constitutive and is unaffected by changes in serum concentration.

While investigating the regulation of FOXM1 function by RAF/MEK/MAPK signaling, we noted another interesting difference between the b and c isoforms (Ma et al., 2005). When a constitutively active form of MEK1 (caMEK1) was coexpressed with either FOXM1c or FOXM1b and the cyclin B1 reporter in transient assays, only FOXM1c showed a significant enhancement of transactivating activity. The enhancing effect was lost when a dominant negative form of MEK1 (dnMEK1) was coexpressed. To explore the biochemical basis of the differential responsiveness of the two isoforms, a FOXM1c or FOXM1b construct, tagged at the C-terminus with a V5 tag, was cotransfected with or without MEK1 (caMEK1 or dnMEK1) into HEK293 cells, and the cell lysates subjected to immunoblot analysis using an anti-V5 antibody (**Figure 2**). Interestingly, the stimulation of RAF/MEK/MAPK signaling was found to promote the proteolytic processing of FOXM1c to generate multiple short forms (**Figure 2A**). One truncated form (indicated by an arrow) migrated with similar mobility as the Del-N form of FOXM1c (artificially generated by the deletion of the N-terminal 187 amino acids). Missing the N-terminal inhibitory domain (Luscher-Firzlaff et al., 2006;Wierstra and Alves, 2006a,b), the Del-N form has long been known to exhibit greatly enhanced transcriptional activity. The proteolytic processing of FOXM1c was suppressed with the coexpression of dnMEK1. However, the proteolytic processing of FOXM1b appeared constitutive and was unaffected by the activation or inhibition of RAF/MEK/MAPK signaling (**Figure 2A**). To confirm this differential regulation of the two isoforms by RAF/MEK/MAPK signaling, we also subjected FOXM1c- or FOXM1b-transfected HEK293 cells to treatment with media containing different amounts of serum. As shown in **Figure 2B**, increases in the serum concentration and therefore in the level of activation of RAF/MEK/MAPK signaling led to the increased processing of FOXM1c, whereas the level of FOXM1b processing was independent of the serum content.

## CONCLUSION AND PERSPECTIVES

In this article, we presented evidence to support the hypothesis that the b isoform, which is frequently overexpressed in cancers, represents a more active form of FOXM1. In contrast, the ubiquitously expressed c isoform is a greatly attenuated form of FOXM1 that requires activation by RAF/MEK/MAPK signaling. It has long been known that FOXM1 is an M phase phosphoprotein and that its phosphorylation is associated with proteolytic processing (Westendorf et al., 1994;Korver et al., 1997) to generate shorter forms of FOXM1. However, it remains unclear whether any of the N-terminal truncated forms, which resembles the Del-N form missing the N-terminal inhibitory domain, represent physiologically functional forms of FOXM1. Phosphorylation by various cyclin/Cdks has previously been shown to relieve the autorepression of FOXM1 by the N-terminal inhibitory domain (Wierstra and Alves, 2006b;Laoukili et al., 2008;Park et al., 2008). Mass spectrometry and further biochemical analyses would be required to determine whether MAPK-activated proteolytic cleavage may represent another regulatory mechanism.

FOXM1c is negatively regulated by RB binding, and the 15-amino-acid domain encoded by exon Va (missing in the b isoform) is within the RB-binding domain. It remains to be tested whether FOXM1b binds with lower affinity to RB and is therefore less repressed. This reduced level of repression might explain why FOXM1b is constitutively processed, whereas FOXM1c might be protected by RB binding. Further, it would be interesting to explore how splicing is regulated in cancer cells to favor increased FOXM1b synthesis. Furthermore, the targeted deletion of exon Va in mice would reveal whether the constitutive expression of FoxM1b will increase the susceptibility of these mice to cancer. The forced expression of FoxM1c by the targeted deletion of the introns flanking exon Va might affect spermatogenesis because the b isoform is highly expressed in the testes, but these mice might be less susceptible to cancer.

In summary, we argue that the further study of the functional and regulatory differences between the b and c isoforms is essential before the therapeutic targeting of FOXM1 in cancer cells can be effective. Further analysis is also necessary to explore the possibility that the targeted activation of FOXM1 expression could be used to enhance cell regeneration without causing cancer. It is worth noting that FoxM1 has recently been shown to regulate Oct4 expression and that its function is required for the maintenance of pluripotency in stem cells (Xie et al., 2010). In addition, FoxM1 has been shown to be an effector of DNA damage response by upregulating the expression of antioxidant genes (Tan et al., 2007;Park et al., 2009). We believe that the positive side of FOXM1 function remains to be exploited.

## Conflict of Interest Statement

The authors declare that the research was conducted in the absence of any commercial or financial relationships that could be construed as a potential conflict of interest.
